# Impact of kangaroo mother care on cerebral blood flow of preterm infants

**DOI:** 10.1186/s13052-014-0083-5

**Published:** 2014-11-13

**Authors:** Afaf A Korraa, Alyaa A I El Nagger, Ragaa Abd El-Salam Mohamed, Noha M Helmy

**Affiliations:** Pediatrics department, Faculty of Medicine for Girls, Al-Azhar University, Cairo, Egypt; Radiology department, faculty of medicine, Al-Azhar University, Cairo, Egypt

**Keywords:** Kangaroo mother care, Premature infants, Cerebral blood flow

## Abstract

**Background:**

Kangaroo mother care (KMC) has been widely used to improve the care of preterms and low birth weight infants. However, very little is known about cerebral hemodynamics responses in preterm infants during KMC intervention. The aim of this study is to evaluate the changes of cerebral blood flow (CBF) in middle cerebral artery, before and after a 30 minute application of KMC in stable preterm infants.

**Methods:**

It is a prospective, pre-post test without a control group study. CBF flow paremeters were measured with Doppler ultrasonography in one middle cerebral artery. Sixty preterm stable infants were assessed before and after 30 min KMC. CBF indices were assessed in different positions before KMC, forty neonates in supine position and 20 in vertical suspension (baby is held vertically away from the skin of his mother). Other dependent variables heart rate and mean arterial blood pressure and Spo_2_ were also studied before and after KMC.

**Results:**

The mean gestational age of the infants was (32 ± 2 weeks), and mean birth weight was (2080 ± 270 gm). Comparing CBF indices (Pulsatility index and Resistive index) before and after KMC has shown a significant decrease in both Pulsatility index (PI) and Resistive index (RI) after 30 min. KMC, the mean values were (2.0 ± 0.43 vs 1.68 ± 0.33 & 0.81 ± 0.05 vs 0.76 ± 0.06 respectively P < 0.05*) with mean difference (0.32 & 95% CI 0.042-0.41 & 0.05 & 95% CI 0.04 to 0.06 respectively P < 0.05*) and increase in end diastolic velocity & mean velocity 30 min of KMC (10.97 ± 4.63 vs. 15.39 ± 5.66 P < 0.05*& 25.66 ± 10.74 vs. 32.86 ± 11.47 P < 0.05* ) with mean difference (− 4.42 & 95% CI −5.67 to −3.18 and −7.21 & 95% CI - 9.41 to 5.00 respectively). These changes indicate improvement in CBF. No correlation has been found between CBF parameters and studied vital signs or SpO2.

**Conclusion:**

Kangaroo mother care improves cerebral blood flow, thus it might influence the structure and promote development of the premature infant's brain.

## Background

In developing countries, around 21% of infant mortality is caused by perinatal conditions. Most of the causes of neonatal death can be prevented or treated through simple, effective and low-cost intervention, at home or in the community [1]*.*

Premature infants in the intensive care environment are exposed to an abnormal environment, repeated invasive procedures and prolonged illness. They are highly susceptible to develop various cerebral lesions like intraventricular hemorrhage or periventricular leukomalacia following cerebral hypoperfusion because of their immature brains. This intense sensory impacts neuro-development and long-term outcomes of the premature infants [2,3].

Kangaroo mother care (KMC) is a method of holding a small nappy neonate in skin-to-skin contact, prone upright on the maternal chest. KMC promotes many health effects for both mother and baby, including but not limited to a decrease in morbidity and mortality, and increase in breastfeeding, weight gain and mother-baby bonding [4,5].

Most of studies on KMC were performed on cardiorespiratory parameters rather than cerebral hemodynamics. Cerebral blood flow is an important parameter in cerebral hemodynamics. It was reported previously in asphyxiated newborn infants and preterm infants; however, no study has yet been performed on the response of cerebral blood flow (CBF) in preterm infants during KMC intervention [6-9].

Spectral Doppler Analysis and Color Flow Mapping have extensively performed in premature neonates to evaluate alterations in cerebral hemodynamics. It allows a better visualization of blood vessels and quantification of cerebral blood flow variations in a given time interval [10].

We aim in the present study to evaluate the effect of KMC on CBF in middle cerebral artery and dependent vital signs in stable preterm infants.

## Methods

### Study design

This is a prospective, pre-post test design study without a control group.

### Population

Sixty healthy clinical stable preterm neonates were included in this study, accessing hemodynamic stable preterms limit the gestational age and birth weight, they were recruited from neonatal units of Al-Azhar and Ain-Shams University Hospitals, during the period from July 2012 to February 2013. Sex, birth weight, mode of delivery, Apgar score were recorded.

They were divided into 2 groups according to their position before application of KMC into: fourty infants in supine position (21 males, 19 females) and twenty infants in vertical suspension (babies were held away from their mother's skin). They were 7 males and 13 females.

Neonates delivered full term or preterm with congenital malformation, severe perinatal complications or central nervous system impairment were excluded from the study. Ethical approval was obtained from the Research and Ethics Committee of Faculty of Medicine, Al Azhar University before commencement. For all eligible neonates, an informed consent was obtained from the parents before enrollment and the following characteristics were recorded: maternal profile like age, antenatal care (ANC) visits, multiple deliveries, risk for sepsis etc.

Neonates were given skin-to-skin contact (KMC) as soon as they became hemodynamically stable, preterm newborns were placed vertically, allowing contact between mother's skin and the skin of the preterm newborn, with his/her head turned sideways, arms flexed and adducted, with the elbows close to the trunk and legs also flexed and adducted. The preterm newborns stayed in the kangaroo position for a minimum of 30 minutes.

All cases were examined when clinically stable, calm and in resting state with no sedation used. Before initiating KMC, physiological parameters (heart rate, blood pressure and oxygen saturation) and cerebral blood flow parameters of middle cerebral artery [end diastolic velocity (EDV) Mean velocity (MV), pulsatility index (PI) and resistive index (RI)] were measured in two different positions (supine & vertical), then these data were collected again immediately after 30 mins of KMC.

Heart rate was measured by using the signal from the Nonin Pulse Oximeter, blood pressure was measured by Dinamap oscillometric monitor.

Middle cerebral artery was visualized by Colored Doppler Ultrasonography (Esaote MyLab apparatus) with a sectorial multi-frequency transducer 7.5 MHz. in the fold of the temporal lobe (as acoustic shadow) from the straight mid portion of the artery while few cases were visualized in frontal lobe. In all cases, the angle between the ultrasound beam and blood flow in the vessel was close to 0 degree, range (0-20°), angle correction and calculation of velocities were made automatically by the instrument's software obtained from five sequential cardiac cycles of optimal quality to obtain reliable flow velocity values [11].

### Statistical analysis

Descriptive data are reported as mean +/− standard deviation, ranges, median and interquartile ranges (IQR). The data collected were statistically analyzed by computer using SPSS release 17 for windows. Tests of statistical significance used were chi-square test, independent t-test, paired t-test (for the physiological parameters) and Pearson correlation coefficients were used to assess the relation between two parameters with quantitative data in the same group. P value of <0.05 was considered as statistically significant.

## Results

Sixty healthy clinical stable preterm neonates, breathing spontaneously without supplemental oxygenation were included in this study. Their mean gestational age was (32 ± 2 weeks), mean postnatal age was 13 ± 4 days and mean birth weight was (2080 ± 270 gm) ranging from 1600 to 2400 gm. Their median Apgar score after one minute was 7 and after 5 minutes was 8.

Comparing data before and after the application of KMC for 30 minutes, there has been a significant decrease in heart rate and an increase in systolic and diastolic blood pressure, MABP and SpO2 (Table 1) .

**Table 1:** **Comparison between vital signs before and after 30 min of KMC**

	**Before KMC N = (60)**	**After KMC N = (60)**		
	**Median (IQR)**	**Median (IQR)**	**t** ^**&**^	**P-value**
**HR (b/min)**	156 (150–159)	151 (146 – 156)	25.008	<0.01*
**SBP (mmHg)**	73 (71 – 74)	74 (74 – 75)	−10.768	<0.01*
**DBP (mmHg)**	32 (31–33)	33 (32 – 33)	−5.333	<0.01*
**MABP**	46 (45–46)	47 (46 – 47)	8.842	< 0.01*
**SpO2 (%)**	95.85 ± 1.4	97.83 ± 1.03	−20.547	<0.01*

HR: Heart rate, SBP: Systolic blood pressure, DBP: Diastolic blood pressure.

MABP: Mean Arterial Blood Pressure.

SpO2 (%): Oxygen saturation.

IQR: Interquartile range.

t^&^: paired t-test, *Significant.

**Figure 1:**
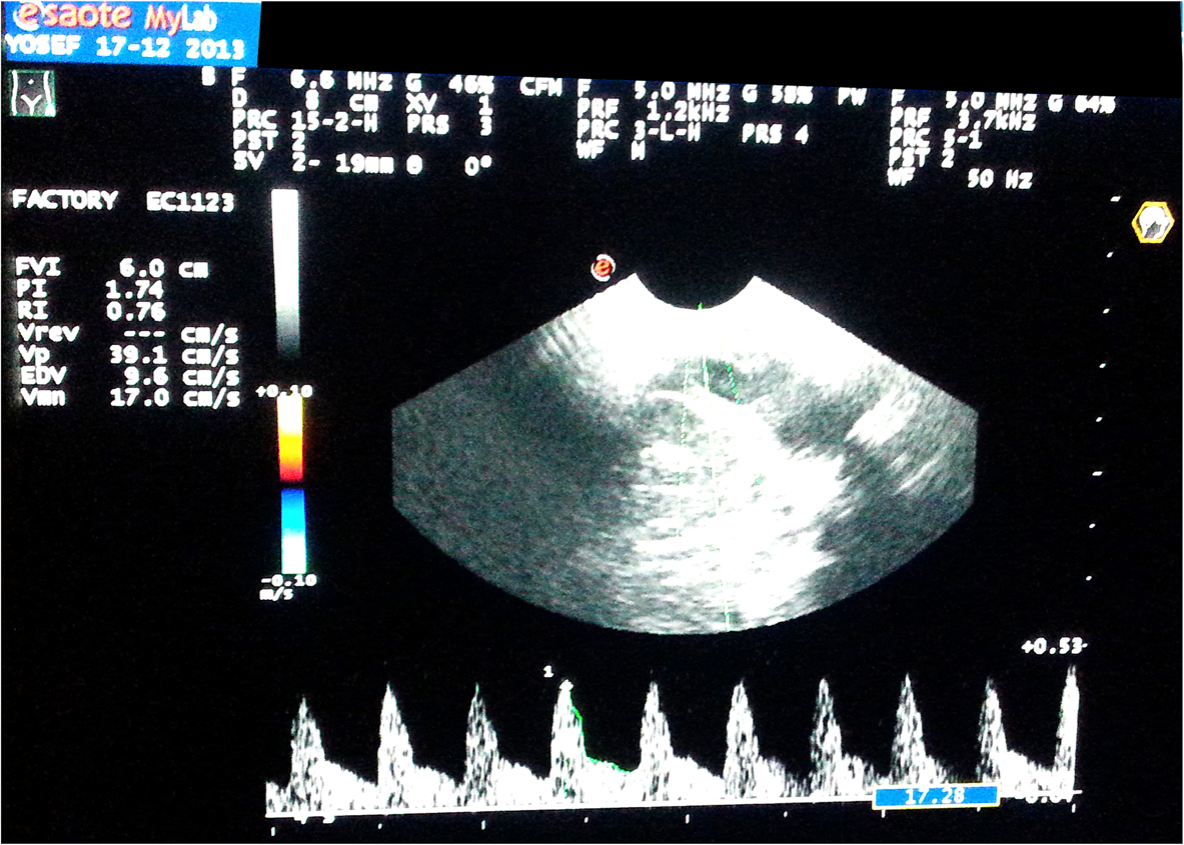
**Colored Doppler Ultrasonography before KMC while the neonate in supine position.**

**Figure 2:**
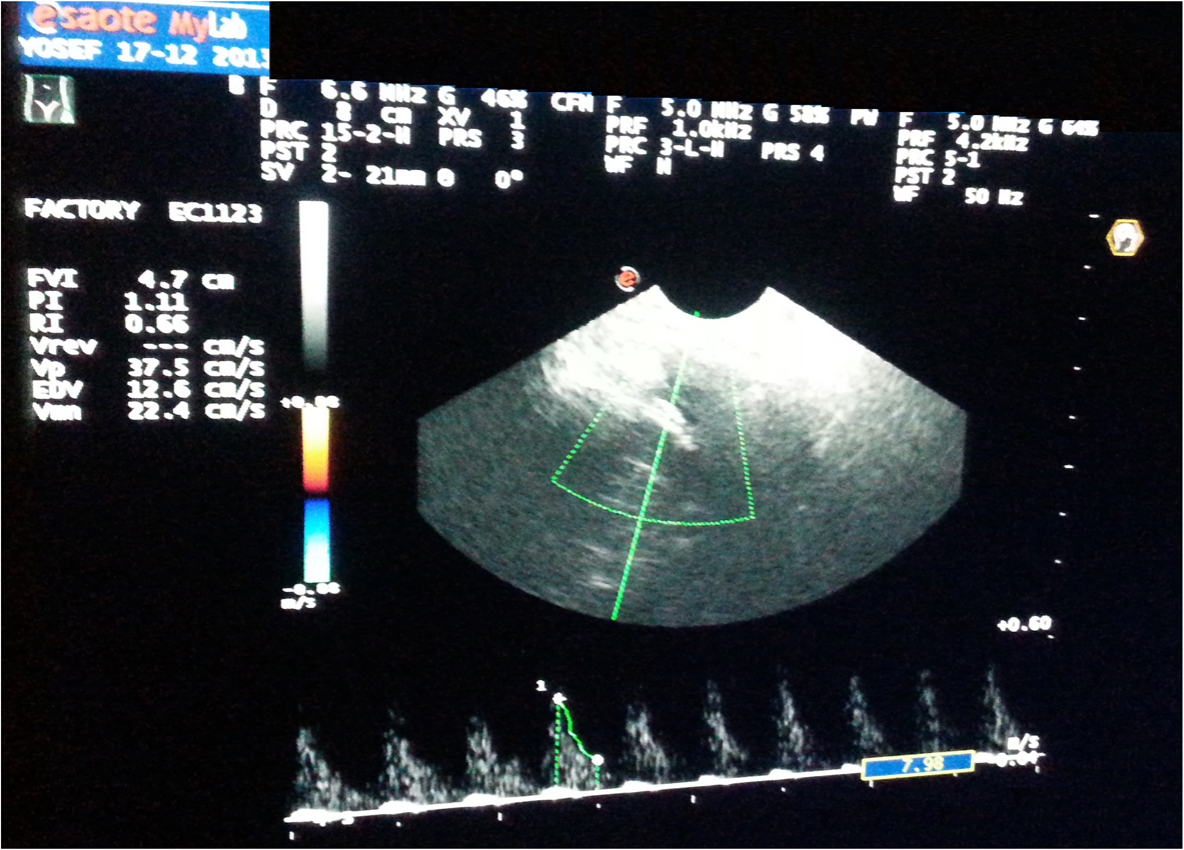
**Colored Doppler Ultrasonography after KMC.**

Regarding CBF parameters, there has been a statistically significant decrease in both PI and RI after 30 min of KMC and increase in the end diastolic velocity and mean velocity (MV) (Table 2, Figures 1 and 2). No significant difference (P >0.05) in CBF parameters between supine and vertical positions before KMC (Table 3). Patients who received the KMC in the supine position evidenced a significant increase in EDV and a decrease in RI at the end of the 30 minutes (Table 4).

**Table 2:** **Comparison between CBF indices before and after 30 min KMC**

	**Before KMC N = (60)**	**After KMC N = (60)**		
	**Mean ± SD**	**Mean ± SD**	**t** ^**&**^	**P-value**
**P.I**	2 ± 0.43	1.68 ± 0.33	7.649	<0.01*
**R.I**	0.81 ± 0.05	0.76 ± 0.06	8.780	<0.01*
**EDV (cm/s)**	10.97 ± 4.63	15.39 ± 5.66	−7.096	<0.01*
**MV (cm/s)**	25.66 ± 10.74	32.86 ± 11.47	−6.549	<0.01*

PI: Pulsatility index, RI: Resistive index.

EDV: End diastolic velocity.

MV: Mean velocity.

t^&^: Paired t-test, *Significant.

**Table 3:** **CBF parameters in different position (Supine &vertical) before KMC**

	**Supine**	**Vertical**		
	**N = 40**	**(n = 20)**		
	**Mean ± SD**	**Mean ± SD**	**t** ^**#**^	**P-value**
**P.I**	2.03 ± 0.45	1.94 ± 0.38	0.78	>0.05
**R.I**	0.82 ± 0.05	0.8 ± 0.05	1.208	>0.05
**EDV **(cm/s)	10.94 ± 4.99	11.03 ± 3.88	0.066	>0.05
**MV** (cm/s)	25.66 ± 11.78	25.65 ± 8.44	0.004	>0.05

PI: Pulsatility index, RI: Resistive index.

EDV: End Diastolic Velocity.

MV: Mean velocity.

t^#^: Independent t-test.

**Table 4:** **CBF parameters in supine position before KMC and 30 min after KMC**

	**Supine before KMC**	**30 min KMC**		
	**N = 40**	**(n = 40)**		
	**Mean ± SD**	**Mean ± SD**	**t** ^**#**^	**P-value**
**P.I**	2.03 ± 0.45	1.7 ± 0.33	3.740	<0.05*
**R.I**	0.82 ± 0.05	0.76 ± 0.07	4.411	<0.01*
**EDV** (cm/s)	10.94 ± 4.99	15.72 ± 6.41	3.722	<0.01*
**MV** (cm/s)	25.66 ± 11.78	31.82 ± 12.1	2.307	0.023

PI: Pulsatility index, RI: Resistive index.

EDV: End diastolic velocity.

MV: Mean velocity.

t^#^: Independent t-tes, *Significant.

## Discussion

Premature infants have significantly more developmental impairment than their term counterparts. Oxygen is regularly used in preterm infants because of their immature lungs and oxygen is important for metabolism and physiological functions [12,13]. This study has shown an improvement in peripheral oxygen saturation (SapO_2_) after 30 min of KMC*.* Researchers have explained the improvement in oxygenation by the fact that the upright position of KMC increases the efficiency of the diaphragm and pulmonary function [14,15]. On other hand, other researchers have concluded no significant changes in oxygen saturation and consumption during KMC [16,17].

In the current study, there has been a statistically significant decrease in heart rate after applying KMC that generally remained within clinically normal range (120–160 bpm). KMC results in significant reduction in B-endorphin as sign of attenuation of stress response. Some researchers have reported an increment in the heart rate during KMC because of changing newborn’s body position from supine to vertical which leads to increased stress for the newborn [18-21] *.*

Regarding blood pressure, our results have shown a significant increase in mean arterial blood pressure after receiving KMC for 30 mins, may be as an infants response to head up tilt by two mechanisms; the vestibular and baroreceptor reflexes. Head up tilt may cause a fast-acting vestibular-mediated increase in BP. Subsequently, a stimulated baroreflex causes falling in HR and BP returns to resting levels. Other studies haven’t shown any significant changes in blood pressure before and after KMC [22-26].

Very little is known about cerebral hemodynamics during KMC intervention. CBF is an important parameter evaluating brain haemodynamics and oxygenation. Moreover, it has been widely used to monitor cerebral perfusion in infants with birth asphyxia or brain lesion. Our results have shown a statistically significant decrease (P < 0.01) in both Pulsatility index and Resisitive index after 30 min of KMC. On the other hand, there has been a statistically significant increase in the end diastolic velocity (EDV) and the mean velocity. These data suggest that kangaroo mother care leads to improvement of CBF within normal physiological range [27-29].

No difference has been observed in calculated cerebral vascular resistance and velocity between supine and vertical (upright) suspension (holding the baby upright and far away from his mother’s skin). On the other hand, when comparing supine position and KMC, we have found a significant increase in cerebral velocities after KMC, this means that skin to skin itself has provoked a considerable increase in CBF velocity irrespective to the upright position. Improvement in CBF has been probably mediated by stabilized cardio respiratory parameters during sleep. Another explanation; is that human hairy skin has slow conducting unmyelinated (C) afferents that respond to touch and skin to skin contact during KMC. Activation of these fibers stimulates the insular cortex (limbic system) to produce mediators (endorphins, neuropeptide and calcitonin gene-related peptide), which in turn enhance postsynaptic Nitric Oxide Synthase. Nitric oxide induces smooth muscle relaxation and plays a pivotal role in regulating blood flow in the microvasculature [30,31].

In our study, blood pressure did not correlate with CBF velocities. The lack of correlation suggests that preterms can already autoregulate their cerebral blood flow during KMC.

Although many aspects regarding the evolutivity of the brain of the preterm infants still need a clarification, the data we reported, if confirmed in further studies, could be extended to lower gestational ages and may be related to the infants’ neurologic outcome to allow a tighter correlation with practice.

## Conclusions

Kangaroo Mother Care improves cerebral blood flow, within its normal level, thus it might positively influence the structure and the development of the premature infant’s brain. Further study is needed to determine the long-term outcomes of the premature infants.

### Limitation in our study

- Accessing haemodynamic stability limits the application of KMC to a higher gestational age (32 +/− 2 weeks) and weight (2080 +/− 270 gm) of studied infants. Even though there is a possibility for extended application of KMC for neonates with lower gestational age and lower birth weight.
- The relatively small sample size.
- There are other advanced ultrasonography that can calculate more measurements.
